# Possibility, relevant similarity, and structural knowledge

**DOI:** 10.1007/s11229-022-03488-2

**Published:** 2022-02-24

**Authors:** Tom Schoonen

**Affiliations:** Centre for Advanced Studies in Humanities “Human Abilities”, Humboldt-Universität zu Berlin, Charlottestraße 81, 10969 Berlin, Germany

**Keywords:** Epistemology of modality, Possibility, Similarity, Relevance, Structural knowledge, Analogy, Causal knowledge

## Abstract

Recently, interest has surged in similarity-based epistemologies of possibility. However, it has been pointed out that the notion of ‘relevant similarity’ is not properly developed in this literature. In this paper, I look at the research done in the field of analogical reasoning, where we find that one of the most promising ways of capturing relevance in similarity reasoning is by relying on the predictive analogy similarity relation. This takes relevant similarity to be based on shared properties that have structural relations to the property of interest. I argue that if we base our epistemology of possibility on similarity reasoning on the predictive analogy similarity relation, we require prior knowledge of the specifics of these structural relations. I discuss a number of possible responses to this on behalf of the similarity theorists given their methodological approach to the epistemology of modality more generally. They could either opt for making explicit the metaphysics underlying these structural relations, in which case they need to spell out how we can come to know these relations. Or they could opt for developing a theory that explains why we do not need to have explicit knowledge of these structural relations; for example by suggesting that we make use of epistemic shortcuts.

## Introduction

Recently, there has been an increased interest in *similarity-based* approaches to the epistemology of possibility (Roca-Royes, 2007, 2017; Hawke, 2011, 2017; Leon, 2017; Dohrn, 2019). Very roughly, similarity theorists hold that knowledge of actuality provides us with justification for our beliefs about what is possible if the objects or events involved are *relevantly similar*.^1^ For example, I have a wine glass of which I believe that it *could* break. According to the similarity theorists, I am justified in having this belief due to the fact that I believe that this wine glass is (relevantly) similar to another wine glass I once had that *did* break. The attractiveness of this view comes from the fact that it is intuitively plausible and promises to ground knowledge of non-actual possibilities in our knowledge of actuality.

We can distil a general description of the crucial similarity reasoning: we know some object, *x*, has a particular property, *P*. From this, we deduce that that same object, *x*, has yet another property, $\Diamond P$ (by the actuality principle: whatever is actual, is possible).^2^ Then, we extrapolate that another, relevantly similar, object, *y*, also has that property, $\Diamond P$. This is the *Similarity Argument*:

**Similarity Argument (SA):**

**P1.**: *x* has property *P*.
**C1.**: *x* has property $\Diamond P$.          (actuality principle)
**P2.**: *x* and *y* are relevantly similar relative to property *P*.^3^
**C2.**: *y* has property $\Diamond P$.          (from C1 and P2)

The crucial premise here is premise 2: *relevant* similarity relative to the property of interest. Let’s call this the *similarity judgement*. This will be the focal point of this paper. A well-known problem for theories of similarity (of any kind, e.g., in semantics of counterfactual conditionals, scientific representations, analogy, etc.) is that “[a]ny two things share infinitely many properties, and fail to share infinitely many others” (Lewis, 1983, p. 346; see also Goodman, 1972; and Morreau, 2010). The challenge this raises for theorists relying on (SA) is that they need to develop a notion of *relevant* similarity that distinguishes between good and bad instances of the similarity argument. For example, my cat and my pillow are both black, both are soft to the touch, both are composed of atoms, *et cetera*. As my pillow is an artefact, I conclude that my cat could also be an artefact. This is clearly not a good instance of similarity reasoning. However, if I conclude based on the fact that my neighbour has broken their leg and that my neighbour and I both are human, have legs, and occasionally engage in sports, that it is possible for me to break my leg, then I seem to have engaged in good similarity reasoning.

The challenge is to give an account of ‘relevance’ that captures the difference between the former and the latter kind of similarity reasoning. According to critics of similarity theories, similarity theorists have failed to do so (cf. Hartl, 2016, p. 286).

In this paper, I contribute to the furthering of similarity-based epistemologies by looking at the research done in the field of analogical reasoning, where we find a broad spectrum of many different kinds of similarity relations that one can use in similarity reasoning (see Gentner, 1983, Gentner & Markman, 1997). A promising way to distinguish between good and bad similarity arguments is by relying on the *predictive analogy* similarity relation (Bartha, 2010). This similarity relation takes *relevant* similarity to be based on shared properties that are structurally related to the property of interest (Sect. 2). I argue that if we base our epistemology of possibility on similarity reasoning reliant on the predictive analogy similarity relation, we require prior knowledge of the specifics of these relations (Sect. 3). Finally, I discuss the consequences this has for similarity-based epistemologies of possibility (Sect. 4).

Before we dive in, let me mention two preliminary points. First, I will focus on the epistemology of (mundane) *possibility* claims (for ordinary agents). An epistemology of possibility is only part of a full-blown epistemology of modality. On top of our beliefs about possibilities, an epistemology of modality also needs to explain our beliefs about necessities. Given the usual interdefinability of possibility and necessity,^4^ interesting questions arise about the relation between the epistemology of possibility and the epistemology of necessity. For example, Hale suggests that there are two *asymmetrical* approaches: “*necessity-based* approaches, which treat knowledge of necessities as more fundamental, and *possibility-based* approaches, which accord priority to knowledge of possibilities” (Hale, 2003, pp. 5–6, original emphases). Alternatively, one might also *reject* either asymmetrical approach and adopt a *symmetrical* approach, where our epistemology of possibility and necessity are (largely) independent of each other (Fischer, 2016, pp. 76–77). Though I will focus on our knowledge of possibility claims, it is important to stress that the work in this paper is compatible with both asymmetrical possibility-based approaches as well as with the epistemology of possibility (as part) of symmetrical approaches.

Secondly, I will assume that all my examples concern *rigid reference* to the objects involved, so we can ignore the philosophical discussions surrounding the *de re* and *de dicto* distinction between the relevant possibility statements (Fitting & Mendelsohn, 1998, p. 213). I will thus use ‘it is possible that this object has this property’ and ‘this object could possibly have this property’ interchangeably.

## Similarity theories and similarities

The similarity argument mentioned above is a particular instance of a more general argument:

**General Similarity Argument (GSA):**

**P1.**: *x* has property *P*.
**P2.**: *x* and *y* are relevantly similar relative to property *P*.
**C2.**: *y* has property *P*.          (from P1 and P2)

We know that a particular object *a* has a particular property, *P*, and we extrapolate that another, relevantly similar, object *b* also has this property. As pointed out, a lot hinges on how one cashes out the notion of ‘relevant similarity’. In this paper, I turn to the vast research that has been done on similarity-based and analogical reasoning (e.g., Hesse, 1966; Gentner, 1983; Helman, 1988; Vosniadou & Ortony, 1989; Falkenhainer et al., 1990; Chalmers et al., 1992; Bartha, 2010, 2019), something that the current literature on similarity-based epistemologies of possibility lacks. I will provide a synthesised overview of the discussions in this field in order to search for an appropriate notion of *relevant* similarity for similarity-based epistemologists of possibility such that the reasoning from (SA) is plausibly cogent.

As I will be talking about ‘similarity’ in many different contexts, let me make some terminological distinctions to keep things clear. First of all, I will use *similarity reasoning* to talk about reasoning that is based on the (general) similarity argument discussed above. Secondly, I will use *similarity judgement* for the judgement of **P2** in ((G)SA). Thirdly, I will use *similarity relation* to talk about the particular relationship between *x* and *y* that grounds or justifies making the similarity judgement. Finally, I will use *similarity theorists* to talk about proponents of similarity-based epistemologies of possibility.

### Domains and analogies

Similarity reasoning is an ampliative method intended to extend one’s knowledge. I will use the phrase ‘domain’ for the source and target of such ampliative reasoning as it is suitably abstract to include concrete objects, situations, hypotheses, complex systems, *et cetera*. In our examples so far, we have focused on similarity reasoning concerning single objects: object *a* is relevantly similar to *b*. However, in general, similarity reasoning might involve complex *domains* with multiple objects: e.g., Rutherford’s analogy between the solar system and the hydrogen atom (cf. Gentner & Jeziorski, 1993). For ease of our discussion, I will focus on *single object domains*—i.e., similarity reasoning involving two (concrete) objects. We call the domain *from* which we reason the *source domain* and the domain *to* which we reason the *target domain*. As I focus on single object domains, I will sometimes use ‘source object’ as a shorthand for ‘object in the source domain’ (and similarly for ‘target object’).

Domains consist of an object (which, in the case of complex domains, may itself consist of multiple objects) and their properties. Of all these properties, some are known to be shared by the objects in the source and target domains; some are known to *not* be shared; and of some it is unknown whether they are shared. We call these sets of properties, respectively, the *positive* analogy, the *negative* analogy, and the *neutral* analogy. The focus of a similarity argument—i.e., the property of the source domain that we are interested in with respect to the target domain – is a subset of the neutral analogy and is called the *hypothetical* analogy (Bartha, 2010, 2019).^5^

We can now say that a similarity judgement helps us to conclude (via a similarity argument) that a particular property holds of the object in the target domain because of some known shared properties with the source object, despite some known properties that differ. Importantly, this is an *epistemic* characterisation in the sense that we focus on those properties that are *known* to be shared, not those that are as a matter of fact shared. Let us consider an example, adapted from Roca-Royes, (2017, p. 226), to make things a bit clearer and to relate the recently introduced terminology to the more general (GSA).

My table, Messy, can support my laptop (i.e., when I place my laptop on Messy’s surface, it doesn’t fall through it). Twin Messy is the table of my colleague. Messy and Twin Messy are both rectangular, both composed of atoms, both are solid, and both are in the same office. Messy is white, yet Twin Messy is black. I have named Messy ‘Messy’, I don’t know if Twin Messy is named. I am curious whether Twin Messy can support my laptop.

The properties ‘being-rectangular’, ‘being-composed-of-atoms’, ‘being-solid’, and ‘being-in-office-F2.08’ are known to be shared by Messy and Twin Messy—i.e., they constitute the positive analogy. Conversely, of the properties ‘being-white’ and ‘being-black’ it is known that they are not shared—i.e., they make up the negative analogy. Of two properties, in this toy example, it is unknown whether they are shared, ‘being-able-to-support-laptop’ and ‘being-named’. These are the neutral analogy and a subset of the neutral analogy is the hypothetical analogy, in this case the property ‘being-able-to-support-laptop’.

### Relevance and vertical relations

The above is just a systematic way of describing similarity reasoning according to (GSA): we find that Messy and Twin Messy share a number of properties and on the basis of this we might conclude that they also share a further property, the hypothetical analogy. However, at this point we are not yet in a position to explain why concluding that Twin Messy can support my laptop constitutes good similarity reasoning. We still haven’t said anything about what constitutes *relevant* similarity.

Here, we can learn from the literature on analogical reasoning. To make it clear what is important for spelling out relevance, they suggest that we should focus on the *relation between the properties* of the object in the source domain (and, correspondingly, of the object in the target domain). We call these relations between the properties of the object in a domain the *vertical relations*. The vertical relations that we are interested in are always *with regards to the hypothetical analogy*. So, if we are interested in whether Twin Messy can support my laptop, then the vertical relations are the relations between (some) properties of Messy and the property of ‘being-able-to-support-laptop’.

At this point, it will be good to briefly focus on *complex* domains that might themselves consist of multiple objects to get things clear (we will return to single object domains after this discussion). When dealing with complex domains, vertical relations are *both* two-place relations *between objects* as well as higher-order relations *between properties* (Gentner, 1983, Gentner & Markman, 1997, Bartha, 2010). For example, when we consider a complex domain that consists of objects in my office, then examples of two-place relations between, e.g., my laptop and the table are ‘being-supported-by’ and ‘being-on-top-of’. However, these tell us nothing yet of the higher-order relations between properties of my laptop (e.g., the fact that the property ‘having-a-full-battery’ is related to ‘being-able-to-turn-on’). In single object domains, vertical relations consist of *only* higher-order relations between properties, so this is not much of an issue; it is when one extends this to complex domains that one has to be careful. For example, Bartha, (2010), who does not draw the distinction between single object and complex domains, is not always clear on what he means when talking of vertical relations as he takes these to be relations “between the objects, relations, and properties within each domain” (p. 14). Yet, it is important to keep two-place relations between objects and higher-order relations between properties distinct, because the latter are of crucial importance for successful similarity reasoning. So, in what follows, I’ll use ‘vertical relations’ to describe the relations between properties of the relevant object (e.g., the relation between Messy’s properties of ‘being-made-of-wood’, ‘being-solid’, and ‘being-able-to-support-laptop’).

In the next section, I will discuss different kind of vertical relations that are proposed by researchers on analogical reasoning. We will see that determining *relevance*, in successful similarity reasoning, relies on interpreting vertical relations in a particular way.

## *Relevant* similarity

Within the literature on analogical and similarity reasoning, it is common practice to distinguish between *surface similarity* and *predictive analogy* as kinds of similarity relations that result in similarity reasoning of different predictive strength. That is, assuming that the similarity judgement holds, the different similarity relations that feature in the similarity judgement *affect* the likelihood that the similarity reasoning has a true conclusion.^6^ The difference between surface similarity and predictive analogy as similarity relations concerns what vertical relations we take to be important.

The surface similarity relation suggests that we can make similarity judgements based on *any* shared properties between the two domains.^7^ In terms of the vertical relations, this boils down to taking the relevant vertical relation to simply be that of ‘co-instantiation’. If we allow for *any* shared properties as being important to the similarity reasoning, we fail to capture any informative structure between the properties in the source domain and the hypothetical analogy; there is no information about what makes the object in the source domain have the hypothetical analogy. That is, there is no sense of *relevance* between the properties for successful similarity reasoning. Given this, it seems clear that surface similarity reasoning cannot help us to distinguish good from bad similarity reasoning (remember the examples concerning the cat/pillow and my/my neighbour’s broken leg).

The question thus becomes, what kind of vertical relations *can* help us ground the justificatory role of similarity reasoning—i.e., what is the right way to cash out relevance? Researchers who focus on analogical and similarity reasoning have converged on the view that we should focus on *informative* higher-order relations between properties. The reason that people reject surface similarity-based similarity reasoning, they point out, is precisely because the surface similarity relation fails to be sensitive to any informative relation between the properties of the object in the source domain and its having the property of the hypothetical analogy (see Hesse, 1966, p. 109; Gentner, 1983, p. 161; Davies, 1988; Russell, 1988; Gentner & Markman, 1997, p. 48; and Bartha, 2010, p. 197).

### Vertical relations as structural relations

Consider the example of determining whether or not a cup could break on the basis of similarity reasoning. What we need to know for successful similarity reasoning is whether the two cups in question share the properties that, in the broken cup, are related to the property ‘breaks’/‘is-broken’ (e.g., ‘being-of-material-*X*’, ‘having-forces-*Y*-acted-upon-it’, and the relations between such properties). The question becomes: what *kind* of relations are generally informative in this sense?

Traditionally, philosophers suggested that we should focus on *causal relations*. For example, Hesse notes that we should think of similarity reasoning “as essentially *a transfer of causal relations* between some characters from one side of the analogy relation to the other” (Hesse, 1966, p. 99, emphasis added). Importantly, it seems that Hesse focuses on *direct causation*—i.e., causation without intervening factors.

Focusing only on direct causation limits the kinds of cases where we can be said to use similarity reasoning. In a contemporary refinement of Hesse’s analysis, Bartha argues that focussing solely on direct causation “is too restrictive” and that we should “replac[e] [the] causal condition with a more general requirement” (2010, pp. 43-44). In order to account for more structural relations than a known direct causation relation, Bartha appeals to Humphreys’ (1981) *aleatory explanations*. Aleatory explanations are explanations based on explanatory relations that are broadly causal and more general than direct causation. They also include explanations that rely on a common cause structure or counteracting causes, i.e., causes “which lower the probability of the effect” (Humphreys, 1981, p. 227). For example, when we say that the cup broke because it fell off the table despite landing on carpet, we cannot analyse this *only* relying on direct causation, but we can with aleatory explanations (Humphreys, 1981, p. 227; Bartha, 2010, p. 114). Aleatory explanations are based on the relations between the properties of the source object *because of which* it has the hypothetical analogy (e.g., Humphreys, 1981, p. 227; Bartha, 2010, p. 114ff). While being more general than direct causation, the relations aleatory explanations are based on still capture the tacit psychological preference we have “for coherence and causal predictive power” in the similarity relations on which our similarity reasoning is based (Gentner & Markman, 1997, p. 47). When it comes to similarity reasoning, the vertical relations that we are interested in include, at least, (Humphreys’) aleatory explanations.^8^

As theories of similarity reasoning are mainly inspired by and appealed to in (the philosophy of) science, they generally fail to take into consideration other metaphysical explanatory relations such as grounding relations, essential relations, mereological relations, *et cetera*. Given that we are interested in the epistemology of modality, we should keep open the possibility that these kinds of metaphysical relations also play a role in similarity-based possibility judgements. In order to include all of these, as well as the aleatory explanations, one may call the relations that we are interested in more generally *structural relations*; allowing one to remain relatively agnostic about exactly what these relations are. Throughout this paper I will use the term ‘structural relations’ to denote all sorts of relations that can in principle be the *because of which* the source object has the hypothetical analogy. This includes direct causal relations, aleatory explanations (i.e. broadly causal), and relations invoked by metaphysical explanations. Even though I remain agnostic about the exact nature of these structural relations, in this paper, in order to simplify the discussion, I will focus on shared aleatory explanations—e.g., causal relations extending beyond direct causes to include common causes, causal chains, counteracting causes, *et cetera*. I do so for two reasons.

First of all, we are concerned with providing a cognitively plausible epistemology of possibility that explains how ordinary people gain knowledge of mundane possibilities (e.g., this coffee cup could break). If we think that in gaining knowledge of such mundane situations we rely on anything like these structural relations in ordinary life, it is unlikely that these involve essential or grounding relations. As Roca-Royes puts it, “[w]e know that my office wooden table can break; [but] it’s not so clear that we know that (whether?) its material origins are essential to it” (Rocasps Royes, 2017, p. 223; Hawke, 2017 makes similar remarks). We do, however, seem to rely on causal relations quite often and are reasonably reliable at reasoning on the basis of such causal relations (cf. Gelman, 2003, Danks, 2009). So, causal reasoning’s place in an explanation of everyday modal judgements is more plausible than reliance on metaphysical explanatory relations such as grounding or essences.

Secondly, these further structural relations are *modally stronger* than causal relations. That is, relations such as grounding, essence, and material constitution are all closely related to pure metaphysical necessity (see for example, respectively, Bliss & Trogdon, 2016, sect. 5; Fine, 1994, Kripke, 1980). What I mean by ‘modally stronger’ is that if something grounds, constitutes, or is the essence of something else, then the relation between these two objects needs to hold in more worlds (namely all metaphysically possible worlds) than when the relation in question is that of causation. The problems that I will raise for similarity reasoning based on the predictive analogy similarity relation concern the modal profile of causation in relation to knowledge of everyday possibilities that it is supposed to be epistemically prior to. These worries all carry over to the (modally) stronger structural relations.^9^

So, a proper similarity relation is one that takes into account the properties that are structurally related to the hypothetical analogy. Similarity judgements based on such a similarity relation make for good similarity reasoning. Phrasing things in terms of *relevant* similarity, this suggests that structural relations (with regards to the hypothetical analogy) determine relevance. Relevant similarity is similarity in terms of the properties that bear a structural relation to the hypothetical analogy.

In the next section, I will argue that these structural relations need to be *known* in order for the conclusion of the similarity reasoning to be justified. If not, then the agent will not be aware if they are reasoning based on the surface similarity or predictive analogy similarity relation—i.e., for all they know, the conclusion may not be justified.

## Predictive analogies and causal knowledge

The goal of this section is to argue that in order to be justified in similarity judgements—i.e., the crucial premise of (GSA) – one needs to have justified beliefs in the structural relations that underwrite the hypothetical analogy in the source object. This means that similarity-based epistemologies of possibility need to explain what justifies the beliefs in the properties *because of which* the source object has the hypothetical analogy. To show this, I will evaluate Bartha’s (2010) account of predictive analogies, the *Articulation Account*, which is the most thoroughly developed philosophical account of the relevant similarity relation. I will argue that on Bartha’s account in order for similarity reasoning to be justified, one requires *prior* justified beliefs (or knowledge) of the structural relations.

### Articulation accounts of predictive analogy

Bartha (2010) develops the *Articulation Account* of analogies and analogical reasoning, which he takes to be “a refinement” (p. 35) of the classical account of Hesse (1966).^10^ On Bartha’s account there are two crucial features of a predictive analogy: *prior association* and the *potential for generalisation*. The prior association is the collection of all those properties that are *known* to be relevant (‘critical’ in Bartha’s terminology) for the hypothetical analogy in the source domain. Bartha notes that prior association is the determination of the source domain having the hypothetical analogy *because of* certain other properties and despite some other properties (Bartha, 2010, p. 114) Definition 4.5.1). The potential for generalisation, roughly, states that it should be known that some of the relevant properties are in the target domain, without any known defeaters (idem, pp. 117-118, Definition 4.5.3).

Epistemically speaking, the articulation model requires the following two step procedure:^11^

1. “*Determine relevance (critical and secondary features)*. [...] [S]ort out which features of the source and target domains are relevant to the conclusion of the argument, and [...] determine their degree of relevance.”
  (Bartha, 2010, p. 102, original emphasis)
2. “*Assess the potential for generalization (plausibility screening)*. The prospects for generalizing the prior association are evaluated by assessing both positive and negative evidence.”    (Bartha, 2010, p. 103, original emphasis)

We can say that in order to be *justified* in believing the conclusion of predictive analogy-based similarity reasoning, we have to determine (i.e., know or justifiably believe) which properties are *relevant* and see if some of these are in the positive analogy, while none of them are in the negative analogy. As Bartha points out, “I maintain that such an argument [i.e., one that satisfies *prior association* and *potential for generalisation*] establishes a relationship of symmetry between the source and target domains. That relationship, [...], implies that we ought to treat the existence of an analogous association in the target domain as a serious possibility” (2010, p. 265). The upshot for similarity reasoning, according to Bartha, is that if we have established that the similarity judgement satisfies the *prior association* and the *potential for generalisation*, then we are justified in accepting the conclusion (Bartha, 2010; 2019, §4.3).

Note that the first step of the epistemological process, determining relevance, is both crucial for one being justified in believing the conclusion *and* requires one to “sort out which features [...] are relevant to the conclusion” (2010, p. 102). According to Bartha “[a]ll identified *contributing causal factors*” are relevant as well as “[a]ll *salient* defeating conditions [...] for these contributing causal factors” (Bartha, 2010, p. 116, first emphasis added). This means that we need to be justified in believing, among other things, what the contributing causal factors are to the having of the property of interest (i.e., the hypothetical analogy). For otherwise we could not determine whether the source and target domain are “like cases [that should] be treated alike” (Bartha, 2010, p. 265).

So, on Bartha’s articulation account of analogies, in order be justified in accepting the conclusion of a similarity judgement we need to determine which features are relevant to the hypothetical analogy. This, in turn, requires us be justified in believing that those properties feature in the relevant structural relations. That is, one needs to know *because of which* properties the source object has the property in question in order for one to be justified in accepting the conclusion of the similarity reasoning.

In the next two subsections, I will discuss two potential worries that one might have regarding my claim that we need to have prior knowledge of the exact structural relations in order to be justified in accepting the conclusion of a similarity argument. The response to the first of these will help clarify exactly what kind of prior knowledge is required.

### Redundancy, determination, and indicators

The first worry one might have is related to one of the problems raised for the Aristotelian analysis of reasoning by similarity. The problem, it has been argued, is that the Aristotelian analysis involves a premise of the form ‘$\forall x (P(x) \Rightarrow Q(x))$’, which seems to trivialise the similarity reasoning.^12^ As Davies points out,

the condition of the similarity *P* being relevant to the conclusion *Q* needs to be weaker than the inheritance rule $\forall x (P(x) \Rightarrow Q(x))$, for then the conclusion in plausible analogies would always follow just by application of the rule to the target. Inspection of the source would then be *redundant*.

Davies, (1988, p. 231, emphasis added)

He defines this as the *Nonredundancy Problem*: the source domain should not be made redundant in an account of similarity reasoning. One may worry that suggesting that similarity reasoning relies on structural vertical relations *also* results in redundancy. The reasoning goes as follows: I know that properties *P* and *R* are structurally related to property *Q* and I know that object *y* has properties *P* and *R*, thus, I conclude, that object *y* has property *Q*. It seems that no source object is needed for this kind of reasoning.^13^ Let me briefly dispel this worry.

First consider Davies’ (1988) suggestion. He suggests we focus on, what he calls, *determination rules*.^14^ These are rules that tell us that certain abstractions of properties are causally related to each other, without specifying the particular instances of these relations (which would make the source redundant). An example from Davies helps to explain things. Consider Sam and Blake, who both own a second-hand truck. Both of their trucks were built in 1986 and are of the brand Cadillac. Assume that we know that Sam’s truck is white and that it is worth $500. Taking the structural relations to be what determines relevance, we can explain that it would be *bad* similarity reasoning to conclude that Blake’s truck would also be white, but it would be *good* similarity reasoning to conclude that Blake’s truck would also be worth, roughly, $500. However, as Davies points out, we do not expect Blake to come to believe that her truck is worth $500 because she has prior knowledge that being a 1986 Cadillac truck causes it to be worth $500; if she did, we would not need Sam’s truck as a source object for the similarity reasoning. The prior knowledge Blake has, Davies suggests, is of the form “the make, model, design, engine-type, condition and year of a car determine its trade-in value” (1988, p. 233). This does not make Sam’s truck (i.e., the source object) redundant, as we need it to justifiably believe a conclusion of a particular instantiation of this causal relation (in this case, that of a 1986 Cadillac truck and the value of $500).

What this shows is that predictive analogical reasoning requires prior knowledge of the relevant structural relations without thereby making the source domain irrelevant (i.e., be relevantly abstract). The structural knowledge required takes the form of aleatory explanations (Humphreys, 1981) and determination rules (Davies, 1988). These capture that the kind of property of which the hypothetical analogy is an instance (e.g., the price of a second-hand truck) is *because of* other properties, some instances of which are in the positive/negative analogy (e.g., year, brand, model, etc.). We need knowledge of the source object to draw conclusions about particular instances of these structural relations. This means that “we must bring a good deal of *prior knowledge* to the situation to tell us whether the conclusions we might draw are justified” (Davies, 1988, p. 228, emphasis added). In particular, for predictive analogical reasoning, we need to explicitly know *what* the structural relations are that are relevant to the hypothetical analogy.

Let’s call the knowledge that would make the source domain irrelevant ‘concrete explicit structural knowledge’. That is, knowledge of the relations between the *determinables* *and* the particular *determinates* in question (e.g., that being a 1986 Cadillac relates to being worth $500). The prior knowledge that is needed to successfully reason by predictive analogy need not be this strong. The relevant prior knowledge needs only to be of the exact relations between the determinables, without the particular instances of the determinates. Call this ‘explicit structural knowledge’ (e.g., knowing that make, model, etc. are related to the trade-in value). Finally, call ‘(mere) structural knowledge,’ knowledge of the fact that there is some sort of structural relation that is relevant, without exactly knowing what the relation is (e.g., not knowing between which properties a structural relation holds).

As I said before, successful similarity reasoning where the relevance is determined as on the predictive analogy theory requires prior *explicit structural knowledge*. This means that we know exactly which and *what kind* of relations hold between the properties in question (e.g., a causal one, one of necessity, etc.). The latter is needed to be able to know that we are *not* concerned with mere surface similarity.

Let me stress that we need to know that there is an *actual* structural relation between the properties involved and that it is not merely a covariation between the properties. Consider the following example to make this clear. In general, the number of people on a train platform covaries with the time it takes for a train to arrive: the more people on the platform, the less time it will take for a train to arrive. However, there is no structural relation between the property of ‘number-people-on-platform’ and ‘time-train-arrives’ in the way this is intended by Humphreys and Bartha; the train does not arrive shortly *because* there are many people on the platform. When you put a large number of people on a platform at 3 o’clock in the morning, it will still take a long time for the first train to arrive. The structural relations that are the ‘because of which’ of the hypothetical analogy are what I’ve called explicit structural relations and are to be distinguished from, e.g., reliable indicators.^15^ Note, however, that if the reliable indicator does feature in the structural relations, e.g., as a *common cause*, then it would be part of the ‘because of which’ and thus would indeed be part of the explicit structural knowledge. In this case, knowledge of the reliable indicators could be (mere) structural knowledge if the agent knows that the relevant properties are ‘somehow’ related; on the other hand, if the agent does know exactly how these indicator properties are related, then they do have explicit structural knowledge.

### Formal accounts of analogy

Another worry that one might have, is that there are other accounts of analogies and similarity reasoning that *don’t* seem to require prior explicit structural knowledge. Formal accounts of analogies are originated in the computational sciences and aim to provide a purely *syntactical* analysis of analogies (e.g., Gentner, 1983; Falkenhainer et al., 1990; Gentner & Markman, 1997; Forbus et al.,^1998^). These theories seem to provide a counterexample to my suggestion that predictive analogical reasoning requires prior explicit structural knowledge. This is because these accounts promise a completely syntactic analysis of predictive analogical reasoning, seemingly without looking at the *content* of the positive, negative, and hypothetical analogies. I will argue that even on the analysis of these theories, predictive analogical reasoning requires prior structural knowledge.

The structure-mapping theory, one of the most well-known computational models of analogies (Chalmers et al., 1992, p. 205), exploits the distinction between properties and higher-order relations between these properties. Based on this distinction, structure-mapping theorists suggest a particular schema for finding successful analogies (Gentner, 1983, p. 158). The first two steps of the schema merely suggest to ignore ordinary property sharing and focus on higher-order relation sharing. The third principle, the Systematicity Principle (henceforth: SP), suggests that we only take into consideration properties that are part of *the largest system of properties* that are related to each other by higher-order relations (Gentner, 1983, pp. 158-164). That is, the relevant properties are those that feature in such a largest system of properties and this system can be determined, supposedly, *independently* of our knowledge of the particular properties involved.

The reliance on prior explicit structural knowledge is obscured by the distinction between the *AI system*, which tries to determine the best analogy mapping, and the *domain-expert*, who hand-codes in all the relevant relations.^16^ The AI system does not look at the content of analogy, but only looks at the relational structures that it is presented with in order to apply the Systematicity Principle. However, here is the crucial part: the saliency of relations is given by a human programmer or subject who *does* know the relevant contents. Our interest here concerns *human* similarity reasoners and, as formal account theorists have admitted, for these the representation is important (Forbus et al., 1998, p. 253). We, as similarity reasoners, are in a sense both the AI system and the domain-expert: we first need to decide which relations we take into account and then consider which ones we map onto the target domain. This first step—the one that mimics the role of the domain-expert—*does* rely on prior explicit structural knowledge.

## Consequences for similarity theories

So far, we have seen that similarity reasoning relies on structural relations in the source object to the hypothetical analogy (Sect. 2) and that in order for one to be *justified* in accepting the conclusion of a similarity argument one needs to have, what I called, explicit structural knowledge—i.e., knowledge of what these structural relations are (Sect. 3.2). That is, being justified on the basis of successful similarity reasoning requires *prior* explicit structural knowledge, which itself needs to be justified, in order to transmit this justification to the possibility claim that we are interested in. This is itself already a significant finding, for it means that if the similarity theorists aim to address the central question of the epistemology of possibility – i.e., how do we *ultimately* acquire knowledge of possibilities—their account hinges on their explanation of how our beliefs about these crucial structural relations are justified. Something that often isn’t explicitly acknowledged.

The question that arises is what the consequences of this are for similarity-based epistemologies of possibility. In this section, I will discuss three ways of moving forward, based on one’s preferred methodological approach to the epistemology of modality in general. The first response suggests that predictive analogy-based similarity-based epistemologies of possibility should focus on addressing a non-central question in the epistemology of modality. The second response is within a *metaphysics-first* outlook on the epistemology of modality, the consequences of which differ depending on one’s preferred metaphysics. Finally, I will consider a potential option for similarity-based epistemologists of possibility who reject both of the preceding methodological approaches to the epistemology of modality.

Let me stress that a lot more can be said about each of the options that I suggest, which I can only treat briefly here. What these brief discussions intend to achieve is to bring the challenges of a similarity-based epistemology to the foreground and raise them as *must-do tasks* for theorists aiming to defend such a theory. I do not intend to suggest that this is an exhaustive list of possible ways in which similarity theorists might respond. But it is a way of categorising, in broad strokes, possible ways forward given some methodological approaches to the epistemology of modality.

### Weak similarity theory

First of all, similarity-based epistemologists of possibility might opt for, what I will call, a *weak similarity theory*. Weak similarity theorists do not aim to address the central question of epistemology of modality, but only focus on the *hierarchical question*—given the distinction between necessity and possibility, is knowledge of one *more* fundamental than knowledge of the others? For these weak similarity theorists, the findings with respect to predictive analogies seem to be an answer to their question: knowledge of possibility is based on prior structural knowledge, so, knowledge of these structural relations has epistemic precedence over knowledge of possibility (and perhaps modality in general).^17^

Even though the weak similarity theory is a logically consistent view that is of some interest, I find it unsatisfyingly modest. I think that similarity theorists should not settle for such a modest approach to the epistemology of modality and that they *should* attempt to address the central question of how we ultimately gain knowledge of possibilities (as Hawke, 2011 and Roca-Royes, 2017 seem to aspire to).

### Causal metaphysics first

Setting aside such weak similarity theories, I will discuss the consequences of my arguments for similarity-based epistemologies of possibility given on one’s methodological approach to the epistemology of modality more broadly. First, I will discuss the consequences for those who adopt a *metaphysics-first* stance and second, in the next subsection, a possible response for those who reject such a methodological approach.

One might, following Mallozzi, adopt a *metaphysics-first* approach to the philosophy of modality, holding that “discussing the nature of *x* before addressing the issue of how we know about *x* is generally a profitable methodology (because an answer to the latter issue largely depends on what *x* is)” (2021, p. S1939). For our purposes, this would suggest that in order to properly evaluate the epistemological consequences of the fact that one needs prior explicit structural knowledge, one has to know the underlying metaphysical relations. That is, putting causal metaphysics first.^18^ The consequences that this has for similarity theorists will (partly) depend on what they take the metaphysics of causation to be.

Similarity theorists taking this option need to (i) spell out what they take the metaphysical relation to be and (ii) explain the appropriate epistemology of these metaphysical relations. In particular, note that the relation between cause and effect differs in modal profile with different accounts of causation. For example, necessitists take there to be a necessary relation between the cause and its effect, whereas regularity theorists deny any modal relation whatsoever. Similarity theorists will have to take this into account when explaining the role this plays in their similarity-based epistemology of *possibility*. Let me make two brief comments on this in order to give a taste of what this option might be like and of the issues that this might raise.

#### Causation as modally-Loaded

First of all, one might take the metaphysical relation of causation to be *modally-loaded*. Two types of theories of the metaphysics of causal relations that involve modal relations are *necessitism* (e.g., Hesse, 1966; Mackie, 1980) and *counterfactual theories* (cf. Lewis, 1973; Collins et al., 2004; Paul, 2009; Menzies & Beebee, 2019). In the case of necessitism it is obvious that the relation between cause and effect is a modal one (namely, necessitation), but even in the case of counterfactual theories this is so. Counterfactual dependence, or counterfactual conditionals, are often evaluated as *variably strict conditionals*. This is a *restricted necessity* analysis of causation: in *all* the closest or nearby possible worlds where the cause is true, the effect is also true. So, even though this is not as strong as necessity *tout court*, it is still a form of necessity. Alternatively, we might focus on the epistemological side of counterfactuals, which suggests we gain knowledge of counterfactuals through engaging with certain imaginative episodes (Williamson, 2007). In this case, we require knowledge of *constitutive facts* (which we hold fixed when we evaluate a counterfactual), which has been argued to also be *modal* (cf. Roca-Royes, 2011; Tahko, 2012).^19^

When providing an epistemology of *possibility*, the reliance on prior knowledge of (restricted) necessity or of constitutive facts is problematic. Either the similarity theorist holds that there is an *asymmetrical* epistemology of modality that is possibility-first and claims that all modal knowledge comes from (or is derivable from) knowledge of possibility. For example, because they think that this is particularly plausible for our modal knowledge concerning concrete objects. In that case, as many have pointed out, when providing an epistemology of possibility (of simple everyday possibility claims), the entire project would be undermined if it relied on prior knowledge of necessities (e.g., Hale, 2003; Roca-Royes, 2011, 2017; Fischer, 2016).

Alternatively, the similarity theorist might hold that the epistemology of modality is *symmetrical*: it might be that there are different approaches—varying in focusing knowledge of possibilities or necessities—for gaining different kinds of modal knowledge. Yet even if one opts for such a symmetrical approach, *within* one particular class of modal claims (e.g., concerning concrete objects), the epistemology thereof is often considered to be uniform. If this were not the case, we would not be able to provide a systematic explanation of how we gain modal knowledge. So, whether or not one believes that the epistemology of modality is asymmetrical, reliance on prior knowledge of necessity within the epistemology of possibility for concrete objects is problematic.

The ‘must-do task’ for similarity theorists who opt for the causal metaphysics first option *and* adopt a metaphysical relation between cause and effect that is modally-loaded, is to defuse these worries. In general, they should be careful that the epistemology of causation, which has epistemic precedence over the epistemology of possibility, does not turn out to be itself reliant on problematic prior modal knowledge.

#### Causation as regularity

One group of theories that explicitly do not rely on any modal relation between the cause and effect are *regularity theories* of causation (Psillos, 2009; Andreas & Guenther, 2021). A crucial element of a regularity theory is that the events that are like the cause in question are regularly followed by (or constantly conjoined with) events that are like the effect in question (Psillos, 2009, p. 131).

The main epistemological issue for such theories is that we seem to be unable to distinguish between genuine causation and coincidences. In particular, for our purposes, the worry is that because a regularity-theorist cannot distinguish between co-instantiations and genuine causation, we lose the benefits of taking ‘relevant similarity’ to be the predictive analogy. We can no longer distinguish causal relations from mere co-instantiation on such a regularity theory and, because of this, it is not clear how the regularity-theorist distinguishes between surface similarities and predictive analogies.^20^

Regularity theorists have developed more sophisticated theories, specifically in order to overcome these issues (see Andreas & Guenther, 2021 for an overview). For example, Mill’s (1882) refinement of Hume’s regularity theory appeals to *laws of nature* in order to distinguish regularities that are merely accidental from those that are ‘truly’ causal. However, there are some worries for such a Millian theory of causation. As Andreas and Guenther note, this introduces a seemingly arbitrary, mind-dependent component: “regularity is determined solely by the world, but the lawlikeness of a regularity rests, in part, on epistemic criteria” (2021, §1.2). If one takes laws of nature to be objective, then these are often taken to be modally stronger than mere co-instantiation and the epistemological question (again) rises how we can come to know what the laws of nature are.

More recently, Strevens (2004) and Baumgartner (2013) have developed (close cousins of) regularity theories of causation. Strevens suggests that causation is to be analysed in terms of causal models, which are used to define a relation of entailment that is supposed to track causality. As he notes, what does the work in his account “is a constraint on causal models that is founded in physical facts about causal influence” and he “assume[s] that these facts can be read off the true theory of everything” (2004, p. 165). The worry for similarity theorists is whether one needs to have prior knowledge of “the true theory of everything” in order to be justified in ordinary, everyday possibility claims (e.g., that this cup can break or that I can catch the bus). Baumgartner, on the other hand, suggests that regularity theories have to satisfy the principle of non-redundancy, which, however, is done by introducing a (weak) form of a necessity condition again (2013, p. 90).

None of the above is supposed to be a knock-down argument against regularity theories (or similarity theories based on them). Rather, it is meant as an illustration of the ‘must-do task’ for similarity theorists who opt for the causal metaphysics first option and take the causal relation *not* to be modally-loaded. These theorists have to develop a theory of causation that is able to overcome the worries of a naïve regularity theory, yet that preserves the epistemological virtues of it in order for be a suitable basis for an epistemology of possibility (i.e., the required prior knowledge should be such that it is intuitively plausible that it has epistemic precedence over our knowledge of possibility).

In general, a similarity theorist who opts for the causal metaphysics first option, has to balance the relation between the modal strength of the relation they take causation to be and the corresponding epistemology very carefully.

### No explicit knowledge

Section 3 concluded that we need to have prior explicit structural knowledge—i.e., knowledge of the exact structural relations that ground the similarity judgement. How might a similarity theorist respond to this if one wants to be more ambitious than weak similarity theorists, yet reject a general metaphysics-first approach? The first thing to note is that explicit structural knowledge is a very strong requirement and we often seem to make justified similarity judgements, even when we might not have prior explicit structural knowledge. Perhaps, one might suggest, we do just as well with *tacit* and *unarticulated*, but reliable awareness of what sort of properties are relevant to the hypothetical analogy.^21^ As with the responses from within the metaphysics-first stance, there are a number of different ways in which one might spell this out. I will mention one way that is inspired by findings from the (cognitive) empirical sciences.

One way to develop this idea would be to weaken the *kind* of knowledge that is required; that is, similarity theorists might suggest a reliance on *epistemic shortcuts*. The suggestion would be that we have knowledge or justified beliefs in something else—i.e., *not* the exact structural relations, but perhaps a placeholder—that allows us to make justified similarity judgements and to justifiably accept the conclusions of similarity arguments.

There is some plausibility to this suggestion. For example, empirical evidence from the classic *triad task* experiments *prima facie* supports this idea (Gelman & Markman, 1986; 1987). In these experiments, agents were asked to compare two comparison objects to a target object. Of these two comparison objects, one would be perceptually similar yet of a different kind and one would be perceptually dissimilar but of the same kind with regards to the target object. For example, if we take a shark as the target object, then two comparison objects could for example be a dolphin and a clown fish. Agents were then asked a variety of ampliative reasoning tasks, all of which showed that the participants favoured category membership over perceptual similarity in such reasoning tasks (Gelman, 2003, p. 30). Importantly, this does *not* require agents to have explicit prior knowledge. With respect to a different example, Gelman points out that “before ever learning about chromosomes or human physiology,” one might believe “that girls have some inner, nonobvious quality that distinguishes them from boys and that generates the many observable differences in appearance and behavior between boys and girls” (2003, p. 9).

This claim has been called the *placeholder heuristic* and is often appealed to in order to explain the data of, e.g., triad tasks (cf. Medin, 1989; Gelman, 2003; and many others). The rough idea is that agents don’t need to know, what we have been calling, the *exact* structural relations, but rely on reasoning with a placeholder. The “claim is that people hold an intuitive belief that [something like exact structural relations] exists, even if its details have not yet been revealed” (Gelman, 2003, p. 10). This is a *heuristic* exactly because the placeholder allows for certain inferences even if the exact details of what make the inferences justified are not known. Relatedly, Danks (2009) points out that “causal inference [relevant to the epistemology of causal relations] is significantly influenced by the categories and concepts that we have (Waldmann & Hagmayer, 2006). People typically do causal inference with the categories that they have prior to learning, *even when those categories are suboptimal for causal inference*” (p. 455, emphasis added).

The epistemic shortcut option exploits our reliance on such concepts or heuristics and suggests that based on justified beliefs with regards to those, we are justified in accepting the conclusions of similarity arguments. The must-do task that remains for the similarity theorists who want to pursue this option, is of course spelling out (i) exactly what it is that features as an epistemic shortcut and (ii) what (epistemic) properties it has so that it can play the role of epistemic shortcut.^22^

## Conclusion

In this paper, I’ve spelled out the kind of similarity reasoning similarity-based epistemologies of possibility rely on and noted that the crucial premise in such arguments is the similarity judgement, which crucially relies on what we take the similarity relation to be. In particular, we need to specify how this similarity relation is constrained if we want the similarity judgement to carry any justificatory force in the similarity argument. If we do not, any two objects can be said to be similar. In terms of the literature on analogical and similarity reasoning, similarity theorists need to specify what they take the vertical relations to be in order to determine *relevance* in relevant similarity.

I have argued that predictive analogies require prior *explicit* structural knowledge, in that the agents need to know the exact structural relations before they can justifiably draw any conclusions from a similarity argument. In general, relying on predictive analogies requires (ambitious) similarity theorists to spell out their views on how they intend to explain this explicit structural knowledge. If they don’t do so, their theory remains explanatorily incomplete and unsatisfactory *as philosophical theories about the justification of our possibility knowledge*. For if they cannot explain how we get justification in the belief concerning the crucial structural relation, they have done little more than explaining one kind of knowledge (modal) with a kind of knowledge that is in equal need of explanation.^23^

I have discussed a number of possible responses on behalf of the similarity theorists. They could either opt for making explicit the metaphysics underlying these structural relations, in which case they need to spell out how we can come to know these relations. Or they could opt for developing a theory that explains why we do not need to have knowledge of these explicit structural relations; for example by suggesting that we make use of epistemic shortcuts.

Exactly which path is best taken by similarity theorists and how they should spell this out, is something that should shape future research on similarity-based epistemologies of possibility.

## Footnotes

1 None of the core issues in the epistemology of modality hinge on the difference between knowledge and justification. So, for the purposes of this paper, I will use ‘knows,’ ‘justifiably believes,’ etc. interchangeably (see also Fischer, 2017, p. 6; Sjölin Wirling, 2021, p. 5655, fn. 1).

2 I will use ‘$\Diamond P$’ as sloppy notation for the property ‘$\lambda z.\Diamond P(z)$’.

3 I am assuming that even though strictly speaking the property of interest is $\Diamond P$, when we engage in this kind of similarity reasoning, we are interested in the properties that are related to *P*. If the target object has these properties, then we know that it *could have (had)* *P* as well—i.e., we know that $\Diamond P$ is true for the target object (in this case *y*).

4 Something is possible just in case its negation is not necessary (in symbols: $\Diamond \phi \equiv \lnot \Box \lnot \phi$) and *vice versa*.

5 Note that in the case of complex domains with multiple objects, it is *not* the case that we are interested in reasoning of the form: ‘all objects in the source domain have property *P*, every object in the source domain is relevantly similar to every object in the target domain, therefore all objects in the target domain have property *P*’. Similarly for the notions of positive, negative, and neutral analogy. I take it that we should view the objects that constitute complex domains to be part of a system or complex object that is the object of the source domain (if phrased in terms of a single object). So a positive analogy of similarity reasoning involving complex domains could be: ‘there is something in the source domain that has property *P*’ and ‘there is a corresponding object in the target domain that has property *P*’.

6 Within this literature, people also often distinguish between, what they call, *anomalies* and *literal similarities* (Gentner, 1983, Gentner & Markman, 1997). The former are instances where the source object and the target object share no properties whatsoever; similarity reasoning based on anomalies obviously fails to be proper justification for its conclusion (even if it happens to be true). It is less clear what the latter is supposed to be. Sometimes, these seem to be cases where the source object *is* the target object (Gentner & Markman, 1997, p. 48, Figure 1), whereas other times it seems to involve sharing a high number of vertical relations as well as first-order properties. If literal similarity is supposed to be identity, then similarity reasoning based on it does not extend one’s knowledge. If, on the other hand, it is supposed to be something weaker, then I take it that the crucial reasoning step hinges on the shared vertical relations involved and most of what I say will carry over.

7 The term ‘surface similarity’ might be misleading in that it suggests the focus on ‘surface’ or ‘observable’ relations, which is not the case. Instead, surface similarity is concerned with any, and only, *first-order*, non-relational, properties.

8 Most influential theorists on analogical and similarity reasoning think that there are no serious competitors to these structural relations when it comes to what makes ordinary similarity reasoning successful.

9 Thanks to an anonymous reviewer for pushing me to include these additional structural relations.

10 The articulation account is very subtle and complex and I can only give a rough, informal overview here (for a complete account see Bartha 2010, especially chapter 4). In particular, Bartha’s model accounts for a whole range of similarity relations *other than* predictive analogies. For example, his model is able to account for mathematical analogies (ch. 5) as well as ‘weaker’ similarity relations such as, what he calls, ‘correlative analogies’ (ch. 4.9 & 6.2). I will leave both of these aside.

11 Bartha (2010) has an additional step that precedes these two: paraphrasing the ‘prior association’ into a particular canonical form. However, for our purposes this step is not important.

12 See Hesse, (1966, ch. 4); Bartha, (2010, ch. 2.2); and Bartha (2019, §3.2) for references to Aristotle’s original work on arguments from likeliness and for the full Aristotelian analysis of which the syllogistic inference is only a part.

13 Thanks to Peter Hawke for pushing me on the redundancy problem for the structural relations.

14 Russell (1988, p. 257) calls these determination rules “causal factors”.

15 Thanks to an anonymous reviewer for suggesting to use the example of indicator properties to make it clearer what I mean by *explicit* structural relations. One way that one might develop the epistemological ‘counterpart’ of this idea is explored in Sect. 4.3 below.

16 Thanks to Arianna Betti for making me aware of this distinction and the implications it has for my argument.

17 What I mean by certain sorts of knowledge having epistemic precedence is the following. If, in order to have justified beliefs or knowledge of *M*, I need prior justified beliefs or knowledge of *C* (i.e., the justified beliefs of *C* are crucial for the transmission of justification involved in getting knowledge of *M*), then knowledge of *C* has epistemic precedence over knowledge of *M*.

18 I focus on the metaphysics of *causal* relations, rather than the structural relations understood more broadly. I do so for ease of exposition and, as mentioned in Sect. 2.1, because many of the other structural relations are modally stronger than the causal relations. I take it that the worries raised here, especially in Sect. 4.2.1 for the modally-loaded interpretations, ultimately carry over to other structural relations.

19 “The distinctive feature of [Williamson’s epistemology] is that it requires us to hold fixed *constitutive facts*. Furthermore, for our counterfactual judgements to amount to counterfactual *knowledge*, it is not enough that we merely happen to hold fixed the right things—our counterfactual judgements would be (extensionally) correct in this case, but hardly knowledge. We need to hold them fixed *knowledgeably*. This seems to require knowledge of what the constitutive facts are. [...] If this is so, [Williamson’s epistemology] implies that [for proper] counterfactual evaluation **we must have prior modal knowledge**. This prior modal knowledge would be a *pre-condition* for counterfactual knowledge” (Roca-Royes, 2011, pp. 548–549, original emphases, boldface added).

20 Note that this is a problem for any Humean account of predictive analogical reasoning; not just for epistemologists of modality.

21 Thanks to an anonymous reviewer for suggesting this worry. I’ve used their phrasing of the problem.

22 For an attempt at spelling out such an approach based on kinds and kind categories, see Schoonen (2020, Ch. 8) and Schoonen (n.d.).

23 This is, I take it, similar to (part of) the sentiment that Roca-Royes expresses when she points out that if an epistemology of possibility relies on prior knowledge of essences, “such an epistemology will not have fully-and-satisfactorily elucidated possibility knowledge *until it has satisfactorily elucidated* essentialist knowledge” (2017, p. 223, emphasis added).
